# Supplementary material for: Protective effects of psychiatric medications against COVID-19 mortality before vaccines
Source: PLoS One. 2025 Feb 24;20(2):e0310438. doi: 10.1371/journal.pone.0310438 (PMC11849848; doi:10.1371/journal.pone.0310438)
Supplement: S1 Table — (DOCX) [file pone.0310438.s001.docx]

| **Variable** | **Type** | **Code** |
| --- | --- | --- |
| COVID-19 | ICD-10-CM Diagnosis | U07.1, U07.2 |
| Schizophrenia |  | F20, F22, F23, F25, F28, F29 |
| Mood |  | F30, F31, F32, F33, F34, F39 |
| Anxiety |  | F40, F41 |
| Hypertension |  | I10, I11, I12, I13, I14, I15, I16, N26.2, I16.74 |
| Diabetes |  | E10, E11, E13 |
| Chronic Kidney Disease |  | I120, I132, E082.2, E11.22, E13.22 |
| Ischemic Heart disease |  | I20, I21, I22, I23, I24, I25 |
| Metabolic Syndrome |  | E88.81 |
| Chronic Obstructive Pulmonary Disease |  | J41, J42, J43, J44, J47 |
| High Body Mass Index |  | Z68.3, Z68.4, Z68.5 |
| Current Smoker |  | Z71.6, Z72.0, Z87.891 |
|  | LONIC | 64234-8 |
|  | CPT | 99406, 99407, G0436, G0437, G9016, S9453, S4995, G9276, G9458, 1034F, 4004F, 4001F, G9906, G9907, G9908, G9909 |
